# Etiologies of Bloody Diarrhea in Children Presenting With Acute Gastroenteritis to US Emergency Departments

**DOI:** 10.1093/ofid/ofae692

**Published:** 2024-11-27

**Authors:** Paola Fonseca-Romero, Ben J Brintz, D Matthew Vierkant, Jennifer Dien Bard, Daniel M Cohen, Ara Festekjian, Amy L Leber, Jami T Jackson, Neena Kanwar, Chari Larsen, Rangaraj Selvarangan, Kimberle C Chapin, Andrew T Pavia, Sharia M Ahmed, Daniel T Leung, the IMPACT study investigators, Andrew T Pavia, Daniel M Cohen, Amy L Leber, Judy A Daly, Jami T Jackson, Rangaraj Selvarangan, Neena Kanwar, Jeffrey M Bender, Jennifer Dien Bard, Ara Festekjian, Susan Duffy, Chari Larsen, Kristen M Holmberg, Tyler Bardsley, Benjamin Haaland, Kevin M Bourzac, Christopher Stockmann, Kimberle C Chapin, Daniel T Leung

**Affiliations:** Department of Pathology, Spencer Fox Eccles School of Medicine, University of Utah, Salt Lake City, Utah, USA; Department of Internal Medicine, Spencer Fox Eccles School of Medicine, University of Utah, Salt Lake City, Utah, USA; Department of Internal Medicine, Spencer Fox Eccles School of Medicine, University of Utah, Salt Lake City, Utah, USA; Department of Internal Medicine, Spencer Fox Eccles School of Medicine, University of Utah, Salt Lake City, Utah, USA; Children's Hospital, Los Angeles, Keck School of Medicine, University of Southern California, Los Angeles, California, USA; Department of Pediatrics, Nationwide Children's Hospital, Columbus, Ohio, USA; Children's Hospital, Los Angeles, Keck School of Medicine, University of Southern California, Los Angeles, California, USA; Department of Pediatrics, Nationwide Children's Hospital, Columbus, Ohio, USA; Children's Mercy Hospital, Kansas City, Missouri, USA; Children's Mercy Hospital, Kansas City, Missouri, USA; Department of Pediatrics, Spencer Fox Eccles School of Medicine, University of Utah, Salt Lake City, Utah, USA; Children's Mercy Hospital, Kansas City, Missouri, USA; Deepull, Barcelona, Spain; Warren Alpert Medical School, Brown University, Providence, Rhode Island, USA; Department of Internal Medicine, Spencer Fox Eccles School of Medicine, University of Utah, Salt Lake City, Utah, USA; Department of Pediatrics, Spencer Fox Eccles School of Medicine, University of Utah, Salt Lake City, Utah, USA; Department of Internal Medicine, Spencer Fox Eccles School of Medicine, University of Utah, Salt Lake City, Utah, USA; Department of Pathology, Spencer Fox Eccles School of Medicine, University of Utah, Salt Lake City, Utah, USA; Department of Internal Medicine, Spencer Fox Eccles School of Medicine, University of Utah, Salt Lake City, Utah, USA

**Keywords:** antibiotic, bloody diarrhea, gastroenteritis, multiplex panel, pediatric

## Abstract

We used molecular testing to examine the causes of bloody diarrhea in a multicenter study of pediatric gastroenteritis. Pathogens typically associated with bloody diarrhea were detected in less than half of cases, and inappropriate antibiotic use was common, supporting the use of stool testing in patients with bloody diarrhea.

Diarrheal diseases continue to be the leading cause of death globally in children under 5, with an estimated 1.7 billion cases and 525 000 deaths every year [1]. In the United States alone, diarrheal illness accounts for 178.8 million cases, resulting in 474 000 hospitalizations and 5000 deaths annually [2]. Bloody diarrhea represents a severe subset, associated with substantial health care utilization and medical costs [3, 4]. Despite this, our knowledge of the causes of bloody diarrhea is limited mainly to single-center studies, many of which were from the premolecular era.

Bloody diarrhea is often associated with *Shigella* spp. and other invasive enteropathogens such as Shiga toxin–producing *E. coli* (STEC), Salmonella spp., *Campylobacter* spp., and *Yersinia* spp. [3, 5–8]. However, recent studies have shown that many cases of bloody diarrhea cannot be attributed to only bacteria, and STEC only accounts for a minority of cases. In many cases, infectious etiology cannot be determined [9, 10]. The Infectious Diseases Society of America guidelines recommend that patients presenting with fever or bloody stool be evaluated for enteropathogens for which antimicrobial agents may be clinically beneficial [11]. Historically, given the long turnaround time of stool culture, the use of antibiotics for bloody diarrhea has been empiric. However, overuse of antimicrobials can cause adverse effects including hemolytic-uremic syndrome (HUS) in STEC infection [12]. On the population level, overuse may lead to the development of antimicrobial resistance. In 1 US study, 13% of children with acute gastroenteritis were prescribed antibiotics at outpatient visits [13]. Knowledge of etiologies in children with bloody diarrhea may improve the appropriate use of antibiotics and diagnostics. Therefore, our primary objective in this study was to determine the etiological agents responsible for bloody diarrhea in children.

## METHODS

We performed a secondary analysis of data collected as part of a pragmatic stepped wedge study of the impact of multiplex polymerase chain reaction (PCR) testing in 5 US pediatric emergency departments (EDs), also known as the Implementation of Molecular Diagnostic for Pediatric Acute Gastroenteritis (IMPACT) study [14], conducted from April 2015 to September 2016. Eligible patients were <18 years old with symptoms of gastroenteritis (diarrhea, vomiting, abdominal pain) who had presented to the ED or on-site urgent care center. As part of the study, during the ED visit or within 48 hours, children were asked to provide a stool sample for PCR testing, regardless of whether testing was ordered for clinical purposes. Multiplex PCR (BioFire FilmArray GI Panel, bioMerieux, Salt Lake City, UT, USA) was performed on all samples collected. In the “pre-intervention” phase of the study, specimens were stored and later tested. Results were not available to clinicians; in the “intervention” phase, specimens were tested in real time at the hospital laboratory, and results were immediately made available to clinicians.

The IMPACT study enrolled 1157 eligible patients (Supplementary Figure 1). Excluding 137 patients who did not have diarrhea or whose status was unknown, among the 1020 (88%) patients who reported diarrhea, we included 126 (12%) who reported bloody diarrhea in this analysis. We considered multiple pathogens detected in a single sample to be co-detection. Given their unclear clinical significance, we did not include detection of enteropathogenic *E. coli* (EPEC) in children of any age or of *C. difficile* in children ≤2 years of age.

We conducted data processing and analyses utilizing R, version 4.2.3. Patient characteristics were summarized using descriptive statistics for 5 age groups. We calculated odds ratios (ORs) and CIs in 2 distinct populations (bloody and nonbloody diarrheal cases) to assess the association between the presence of a pathogen and the likelihood of experiencing bloody diarrhea. In addition, we calculated OR adjusted by age group.

## RESULTS

Among 1020 patients <18 years old with diarrhea, 126 (12%) children or their caregivers reported bloody diarrhea (Supplementary Figure 1). Multiplex PCR was performed on all subjects with submitted stool samples, which included 111 (88%) children with bloody diarrhea; at least 1 pathogen was detected in 69 (62%). We detected viruses in 20 (18%), bacteria in 67 (60%), and protozoa (*Giardia* only) in 2 (2%) of the 111 children with bloody diarrhea who underwent PCR testing (Table 1; Supplementary Table 1). Pathogens typically seen in bloody diarrhea cases, including *Campylobacter*, *C. difficile*, STEC, and *Shigella*, were detected in 46 (41%) of the cases. These include either single detection or co-detections with multiple pathogens. Interestingly, pathogens were not detected in 42 (38%) children. In the 52 (47%) patients where only 1 pathogen was detected, we identified bacteria in 36 (69%), viruses in 14 (27%), and protozoa (*Giardia* only) in 2 (4%) (Supplementary Table 2).

**Table 1.** Frequency of Detection for the 21 Pathogens in the PCR Panel Among Patients Presenting With Bloody Diarrhea, Stratified by Age Group

Pathogen	<6 Months (n = 12), No. (%)	6–23 Months (n = 23), No. (%)	2–4 Years (n = 19), No. (%)	5–11 Years (n = 34), No. (%)	12–17 Years (n = 23), No. (%)	Total (n = 111), No. (%)
Bacteria	…	…	…	…	…	n = 67
*Campylobacter*	0 (0)	2 (9)	4 (21)	1 (3)	1 (4)	8 (7)
*C. difficile*	0 (0)	0 (0)	3 (16)	3 (9)	3 (13)	9 (8)
ETEC	0 (0)	0 (0)	1 (5)	3 (9)	0 (0)	4 (3.6)
EAEC	0 (0)	1 (4)	2 (11)	7 (21)	0 (0)	10 (9)
*Salmonella*	2 (17)	0 (0)	1 (5)	3 (9)	0 (0)	6 (5.4)
*P. shigelloides*	0 (0)	0 (0)	0 (0)	0 (0)	1 (4)	1 (0.9)
*Vibrio*	0 (0)	0 (0)	0 (0)	0 (0)	0 (0)	0 (0)
STEC	0 (0)	3 (13)	2 (11)	4 (12)	5 (22)	14 (13)
O157	0 (0)	1 (4)	1 (5)	1 (3)	2 (9)	5 (4.5)
Non-O157	0 (0)	2 (9)	1 (5)	3 (9)	3 (13)	9 (8)
*Shigella/*EIEC	0 (0)	2 (9)	2 (11)	10 (29)	1 (4)	15 (14)
*Y. enterocolitica*	0 (0)	0 (0)	0 (0)	0 (0)	0 (0)	0 (0)
*V. cholerae*	0 (0)	0 (0)	0 (0)	0 (0)	0 (0)	0 (0)
Viruses	…	…	…	…	…	n = 20
Adenovirus F	0 (0)	0 (0)	0 (0)	0 (0)	0 (0)	0 (0)
Astrovirus	1 (8)	2 (9)	1 (5)	0 (0)	0 (0)	4 (3.6)
Norovirus	1 (8)	1 (4)	0 (0)	1 (3)	2 (9)	5 (4.5)
Sapovirus	1 (8)	4 (17)	1 (5)	0 (0)	0 (0)	6 (5.4)
Rotavirus A	4 (33)	1 (4)	0 (0)	0 (0)	0 (0)	5 (4.5)
Protozoa	…	…	…	…	…	n = 2
*Giardia*	0 (0)	0 (0)	1 (5)	1 (3)	0 (0)	2 (1.8)
*Cryptosporidium*	0 (0)	0 (0)	0 (0)	0 (0)	0 (0)	0 (0)
*Cyclospora*	0 (0)	0 (0)	0 (0)	0 (0)	0 (0)	0 (0)
*E. histolytica*	0 (0)	0 (0)	0 (0)	0 (0)	0 (0)	0 (0)
No pathogen detected	4 (33)	11 (48)	5 (26)	10 (29)	12 (52)	42 (38)

Co-occurring pathogens are listed multiple times. *Vibrio* spp., *V. cholerae*, *E. histolytica*, *Y. enterocolitica*, adenovirus F 40/41, *C. cayetanensis*, and cryptosporidium were not detected.

Abbreviations: EAEC, enteroaggregative *E. coli*; ETEC, enterotoxigenic *E. coli*; O157, *E. coli* O157; STEC, Shiga toxin–producing *E. coli*.

Those with bloody diarrhea had a higher mean and median age (6.5 and 5.2 years, respectively), compared with the overall group (4.7 and 2.5 years) (Supplementary Table 3). A large proportion of those with bloody diarrhea were aged 5–11 years (31%), compared with 22% of all diarrhea patients. Other notable differences include the increased prevalence of international travel in the bloody diarrhea group.

We analyzed the association between the presence of pathogens and bloody diarrhea (Table 2), comparing patients with bloody diarrhea (n = 111) with those with nonbloody diarrhea (n = 731). The odds of having bloody diarrhea were 9.4 times higher in individuals with STEC detected than those without (unadjusted OR, 9.4; 95% CI, 4.2–21.9). After adjusting for age, the odds remained substantially elevated, with an adjusted OR of 9.0 (95% CI, 3.9–21.0). Similarly, the odds ratio for *Campylobacter* was 4.3 (95% CI, 1.7–10.4). In contrast, those with sapovirus, astrovirus, and norovirus were less likely to have bloody diarrhea, although the confidence interval excluded 1 only for norovirus and sapovirus.

**Table 2.** Unadjusted and Age Group–Adjusted Odds Ratios of the Association Between the Presence of Pathogen and the Occurrence of Bloody Diarrhea

Pathogen^a^	Bloody Diarrhea With PCR (n = 111), No. (%)	Nonbloody Diarrhea With PCR (n = 731), No. (%)	Unadj. OR (95% CI)	Adjust. OR^c^ 95%CI
STEC	14 (13)	11 (1.5)	**9.4** (4.2–21.9)	**9.0** (3.9–21.0)
O157	5 (4.5)	1 (0.1)	**34.4** (5.5–663.1)	**30.0** (4.6–574.8)
Non-O157	9 (8.1)	10 (1.4)	**6.4** (2.5–16.0)	**6.3** (2.4–16.0)
*P. shigelloides*	1 (0.9)	1 (0.1)	6.6 (0.3–168.6)	4.9 (0.19–127.7)
*Campylobacter*	8 (7.2)	13 (1.8)	**4.3** (1.7–10.4)	**3.9** (1.5–9.5)
*C. difficile*	9 (8.1)	29 (4.0)	**3.9** (1.5–9.5)	1.6 (0.7–3.4)
ETEC	4 (3.6)	9 (1.2)	3.0 (0.8–9.4)	2.8 (0.8–9.0)
*Shigella/*EIEC	15 (14)	39 (5.3)	**2.8** (1.4–5.1)	**2.5** (1.3–4.6)
*Salmonella*	6 (5.4)	22 (3.0)	1.8 (0.7–4.4)	1.7 (0.6–4.0)
EAEC	10 (9.0)	42 (5.7)	1.6 (0.7–3.2)	1.8 (0.8–3.6)
Rotavirus A	5 (4.5)	23 (3.1)	1.5 (0.5–3.6)	2.0 (0.7–5.3)
*G. lamblia*	2 (1.8)	14 (1.9)	0.94 (0.1–3.4)	0.8 (0.1–3.1)
Astrovirus	4 (3.6)	38 (5.2)	0.7 (0.2–1.7)	0.8 (0.2–2.1)
Sapovirus	6 (5.4)	82 (11)	0.5 (0.17–0.98)	0.5 (0.2–1.1)
Norovirus	5 (4.5)	176 (24)	**0.15** (0.05–0.34)	**0.2** (0.05–0.3)
Adenovirus F	0 (0)	92 (13)	^b^	^b^
*Cryptosporidium*	0 (0)	23 (3.1)	^b^	^b^
*Y. enterocolitica*	0 (0)	2 (0.3)	^b^	^b^

Bolded odds ratios are ones that have estimates with 95% confidence intervals (CI) that don’t include 1.0. Abbreviations: Adjust., adjusted; OR odds ratio; Unadj., unadjusted.

^a^No detection of *Vibrio* spp., *V. cholerae*, *E. histolytica*, or *C. cayetanensis* in either group.

^b^No detection of adenovirus F, *Y. enterocolitica*, or cryptosporidium in cases of bloody diarrhea.

^c^Odds ratio adjusted by age category.

Among the 26 children with bloody diarrhea with PCR who initiated antibiotics during or after their emergency department visit (Supplementary Table 4), 10 (38%) had no pathogen for which antibiotics were indicated; 6 (23%) patients had no pathogen or only a virus detected, 2 had *Salmonella* detected in a noninfant, and 2 had STEC, for which antibiotics are potentially harmful.

## DISCUSSION

Bloody diarrhea is thought to be a sign of invasive enteric infection that carries the risk of severe morbidity and mortality among children [8, 9]. Before the widespread availability of multiplex PCR, studies of bloody diarrhea in children had been limited to conventional stool culture, immunoassays, and a limited array of PCR detection methods [3, 5, 6]. Our analysis of multiplex PCR data from a multisite US-based study revealed that pathogens typically associated with bloody diarrhea were detected in fewer than half of these cases. In a substantial proportion (over a third) of bloody diarrhea cases, a pathogen was detected for which antibiotics were not indicated.

Our findings align with more contemporary multiplex PCR-based studies of either bloody diarrhea or, more generally, hematochezia, emphasizing the complexity of attributing bloody diarrhea symptoms to specific pathogens [9, 10]. In our study, 27% (14/52) of children with bloody diarrhea had only viral detection. We also did not identify any pathogens in 38% of the patients, suggesting that some bloody diarrhea cases could be attributed to noninfectious causes or a diarrheal pathogen that is not included among the targets of the panel. In addition, we found that 35% of patients with bloody diarrhea who received antibiotics during or after the ED visit did not have a pathogen detected for which an antibiotic would be indicated; 15% had a pathogen for which antibiotics may be harmful. These findings suggest that molecular testing, such as multiplex PCR, with increased sensitivity for pathogen detection as well as faster turnaround time, may be useful in patients with bloody diarrhea to guide antibiotic use instead of relying on symptom-based empiric therapy. However, more work is needed to identify strategies for optimal stewardship of this resource.

While our study provides valuable insights into the etiologies associated with bloody diarrhea in children presenting to emergency departments in the United States, several limitations warrant consideration. The lack of control groups of nondiarrheal patients limits the ability to draw definitive conclusions about causation between identified pathogens and bloody diarrhea. Second, in 1 of the 5 sites, a *Shigella* outbreak occurred during the study, which could lead to this pathogen being over-represented in our study. Third, our study only included patients presenting to emergency departments and thus may represent more severe cases, restricting the generalizability of our findings. Lastly, no further investigation was conducted to determine the cause of bloody stools in children where no pathogen or atypical pathogens (viruses) were identified. Bloody diarrhea was determined by caregiver report, which may be inaccurate, or attributable to rectal tear or perirectal irritation and not the infectious agent; however, this limitation is common among studies of bloody diarrhea. Despite these limitations, the study underscores the need for comprehensive testing and improved data collection strategies to improve antibiotic stewardship and patient care.

In conclusion, we found that fewer than half of the 12% of participants in this study of children presenting to US-based emergency rooms with acute diarrhea who reported bloody diarrhea had bacterial pathogens typical for such. Recognizing the heterogeneity of etiological agents in bloody diarrhea is crucial to appropriately managing these children.

## Supplementary Material

ofae692_Supplementary_Data

## Supplementary Data

Supplementary materials are available at *Open Forum Infectious Diseases* online. Consisting of data provided by the authors to benefit the reader, the posted materials are not copyedited and are the sole responsibility of the authors, so questions or comments should be addressed to the corresponding author.
