# Supplementary material for: Etiologies of Bloody Diarrhea in Children Presenting With Acute Gastroenteritis to US Emergency Departments
Source: Open Forum Infect Dis. 2024 Nov 27;11(12):ofae692. doi: 10.1093/ofid/ofae692 (PMC11651156; doi:10.1093/ofid/ofae692)
Supplement: ofae692_Supplementary_Data [file ofae692_supplementary_data.pdf]

Supplemental Materials

Etiologies of bloody diarrhea in children presenting with acute gastroenteritis to US emergency departments

Paola Fonseca-Romero[1,2], Ben J. Brintz Ph.D[2], D. Matthew Vierkant[2], Jennifer Dien Bard Ph.D[3], Daniel M. Cohen MD[5], Ara Festekjian MD, MS[3], Amy L. Leber PhD[5], Jami T. Jackson DO[6], Neena Kanwar Ph.D, MVSc[6], Chari Larsen MD[4], Rangaraj Selvarangan Ph.D[6], Kimberle C. Chapin[7,8], Andrew T. Pavia MD[2,4*], Sharia M. Ahmed Ph.D[2*], and Daniel T. Leung MD, MSc.[1,2*] On behalf of IMPACT study investigators

* co-senior authors

Author Affiliations:

[1]Department of Pathology, Spencer Fox Eccles School of Medicine, University of Utah, Salt Lake City, UT, USA

[2]Department of Internal Medicine, Spencer Fox Eccles School of Medicine, University of Utah, Salt Lake City, UT, USA

[3]Children's Hospital, Los Angeles; Keck School of Medicine, University of Southern California, Los Angeles, CA, US

[4]Department of Pediatrics, Spencer Fox Eccles School of Medicine, University of Utah, Salt Lake City, UT, USA

[5]Department of Pediatrics, Nationwide Children's Hospital, Columbus, OH, USA

[6]Children's Mercy Hospital, Kansas City, MO, USA

[7]Deepull, Barcelona, Spain

[8]Warren Alpert Medical School, Brown University, Providence, RI, USA

Corresponding Author: Daniel T. Leung, MD, MSc, FIDSA, Daniel.Leung@utah.edu

Supplemental Figure 1. Flowchart of data from IMPACT study [15] included for analysis.

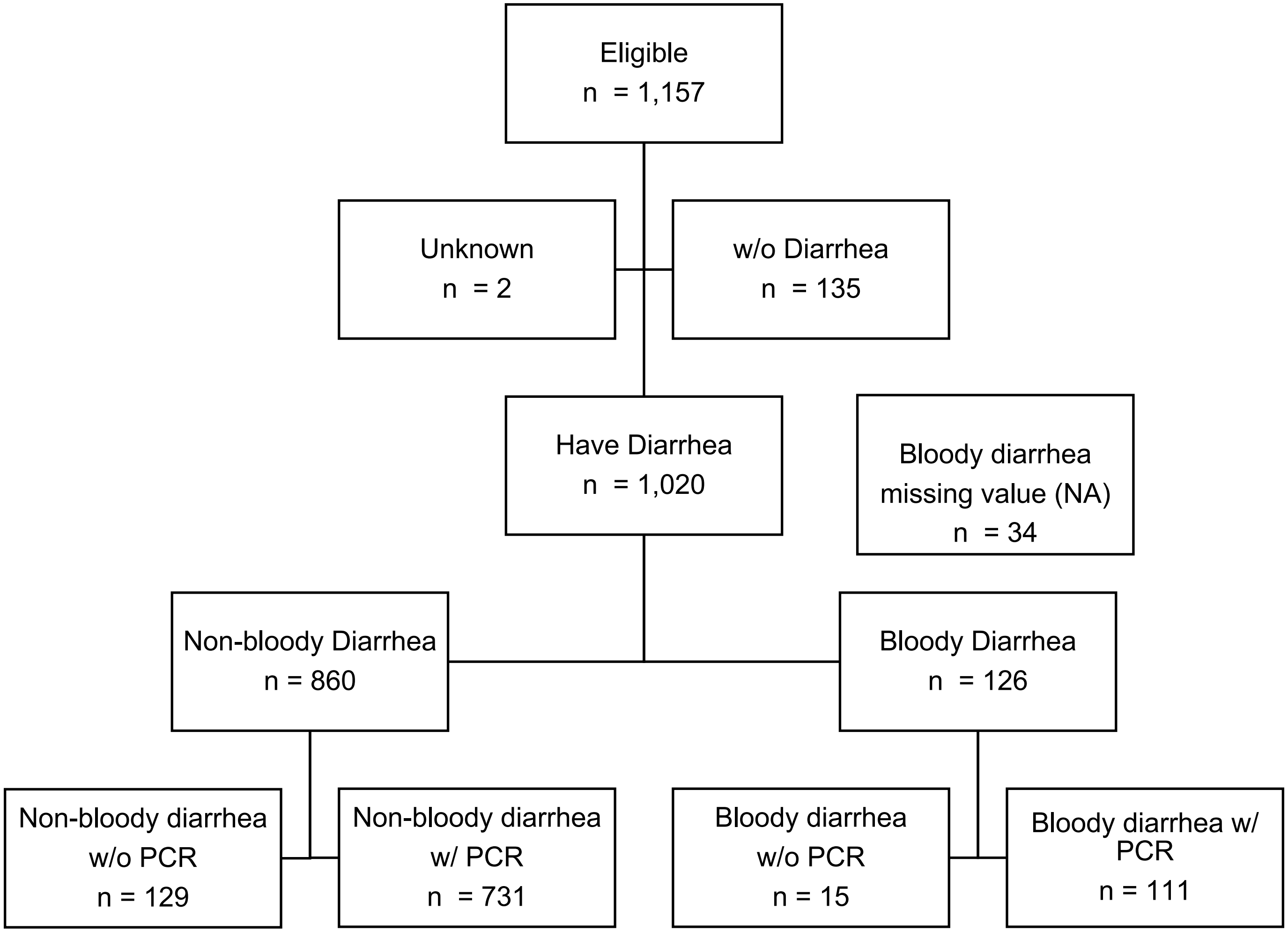

Supplemental Table 1. Frequency of detection for the 21 pathogens in the PCR panel, among patients presenting with bloody diarrhea, stratified by age group. Co-occurring pathogens are listed multiple times. Abbreviations: ETEC, Enterotoxigenic *E. coli*; EAEC, Enteroaggregative *E. coli*; STEC, Shiga toxin-producing *E. coli*; O157, *E. coli* O157

**Characteristics**	**PHC (n = 26)**	**RIH (n = 27)**	**NCH (n = 26)**	**CHLA (n = 13)**	**CMH (n = 19)**	**Total (n =111)**
**Bacteria**						**n = 67**
Campylobacter	3 (12%)	2 (7%)	1 (4%)	1 (8%)	1 (5%)	8 (7%)
C. difficile	1 (4%)	2 (7%)	2 (8%)	0 (0%)	4 (21%)	9 (8%)
ETEC	2 (8%)	0 (0%)	1 (4%)	1 (8%)	0 (0%)	4 (3.6%)
EAEC	2 (8%)	1 (4%)	4 (15%)	1 (8%)	2 (11%)	10 (9%)
Salmonella	1 (4%)	3 (11%)	2 (8%)	0 (0%)	0 (0%)	6 (5.4%)
P. shigelloides	0 (0%)	1 (4%)	0 (0%)	0 (0%)	0 (0%)	1 (0.9%)
Vibrio	0 (0%)	0 (0%)	0 (0%)	0 (0%)	0 (0%)	0 (0%)
STEC	5 (19%)	2 (8%)	6 (23%)	0 (0%)	1 (5%)	14 (13%)
O157	1 (4%)	1 (4%)	3 (12%)	0 (0%)	0 (0%)	5 (4.5%)
Non-O157	4 (15%)	1 (4%)	3 (12%)	0 (0%)	1 (6%)	9 (8%)
*Shigella/*EIEC	1 (4%)	0 (0%)	1 (4%)	0 (0%)	13 (68%)	15 (14%)
Y. enterocolitica	0 (0%)	0 (0%)	0 (0%)	0 (0%)	0 (0%)	0 (0%)
V. cholerae	0 (0%)	0 (0%)	0 (0%)	0 (0%)	0 (0%)	0 (0%)
**Viruses**						**n = 20**
Adenovirus. F	0 (0%)	0 (0%)	0 (0%)	0 (0%)	0 (0%)	0 (0%)

Astrovirus	2 (8%)	1 (4%)	1(4%)	0 (0%)	0 (0%)	4 (3.6%)
Norovirus	1 (4%)	3 (11%)	1 (4%)	0 (0%)	0 (0%)	5 (4.5%)
Sapovirus	1 (4%)	1 (4%)	4 (15%)	0 (0%)	0 (0%)	6 (5.4%)
Rotavirus. A	1 (4%)	3 (11%)	1 (4%)	0 (0%)	0 (0%)	5 (4.5%)
**Protozoa**						**n = 2**
Giardia	0 (0%)	0 (0%)	2 (8%)	0 (0%)	0 (0%)	2 (1.8%)
Cryptosporidium	0 (0%)	0 (0%)	0 (0%)	0 (0%)	0 (0%)	0 (0%)
Cyclospora	0 (0%)	0 (0%)	0 (0%)	0 (0%)	0 (0%)	0 (0%)
E. histolytica	0 (0%)	0 (0%)	0 (0%)	0 (0%)	0 (0%)	0 (0%)
**Age Group**						
<6 months	3 (12%)	6 (22%)	3 (12%)	0 (0%)	0 (0%)	12 (11%)
6-23 months	7 (27%)	4 (15%)	8 (31%)	2 (15%)	2 (11%)	23 (21%)
2-4 years	4 (15%)	3 (11%)	5 (19%)	1 (15%)	5 (26%)	19 (17%)
5-11 years	10 (38%)	7 (26%)	3 (12%)	4 (31%)	10 (53%)	34 (31%)
12-17 years	2 (8%)	7 (26%)	7 (27%)	5 (38%)	2 (11%)	23 (21%)
**No pathogen detected**	12 (46%)	11 (41%)	7 (27%)	11(85%)	1 (5%)	42 (38%)

Footnote: *Vibrio spp*, *V. cholerae, E. histolytica*, *Y. enterocolitica,* Adenovirus F 40/41, *C. cayetanensis,* and Cryptosporidium were not detected. Abbreviations: PHC, Primary Children's Hospital; RIH, Rhode Island Hospital; NCH, National Children's Hospital; CHLA, Children's Hospital of LA; CMH, Children's Mercy Hospital

Supplemental Table 2. Frequency table for each of the 21 pathogens tested in the PCR panel, in cases where only one pathogen was detected.

Pathogen	<6 months (n = 7)	6-23 months (n = 8)	2-4 years (n = 11)	5-11 years (n = 17)	12-17 years (n = 9)	Total (n = 52)
**Bacteria**						**n = 45**
Campylobacter	0 (0%)	1 (13%)	3 (27%)	0 (0%)	0 (0%)	4 (8%)
C. difficile	0 (0%)	0 (0%)	2 (18%)	2 (12%)	2 (22%)	6 (12%)
ETEC	0 (0%)	0 (0%)	0 (0%)	0 (0%)	0 (0%)	0 (0%)
EAEC	0 (0%)	0 (0%)	0 (0%)	2 (12%)	0 (0%)	2 (4%)
Salmonella	1 (14%)	0 (0%)	1 (9%)	2 (12%)	0 (0%)	4 (8%)
P. shigelloides	0 (0%)	0 (0%)	0 (0%)	0 (0%)	0 (0%)	0 (0%)
Vibrio	0 (0%)	0 (0%)	0 (0%)	0 (0%)	0 (0%)	0 (0%)
STEC	0 (0%)	1 (13%)	1 (9%)	2 (12%)	5 (56%)	9 (17%)
O157	0 (0%)	0 (0%)	1 (9%)	1 (6%)	2 (22%)	4 (8%)
Non-O157	0 (0%)	1 (13%)	0 (0%)	1 (6%)	3 (33%)	5 (10%)
*Shigella/*EIEC	0 (0%)	2 (25%)	1 (9%)	7 (41%)	1 (11%)	11 (21%)
Y. enterocolitica	0 (0%)	0 (0%)	0 (0%)	0 (0%)	0 (0%)	0 (0%)
V. cholerae	0 (0%)	0 (0%)	0 (0%)	0 (0%)	0 (0%)	0 (0%)
**Viruses**						**n = 14**
Adenovirus. F	0 (0%)	0 (0%)	0 (0%)	0 (0%)	0 (0%)	0 (0%)
Astrovirus	1 (14%)	0 (0%)	1 (9%)	0(0%)	0 (0%)	2 (4%)
Norovirus	1 (14%)	1 (13%)	0 (0%)	1 (6%)	1 (11%)	4 (8%)
Sapovirus	1 (14%)	2 (25%)	1 (9%)	0 (0%)	0 (0%)	4 (8%)
Rotavirus. A	3 (43%)	1 (13%)	0 (0%)	0 (0%)	0 (0%)	4 (8%)
**Protozoa**						**n = 2**
Giardia	0 (0%)	0 (0%)	1(9%)	1 (6%)	0 (0%)	2 (4%)
Cryptosporidium	0 (0%)	0 (0%)	0 (0%)	0 (0%)	0 (0%)	0 (0%)
Cyclospora	0 (0%)	0 (0%)	0 (0%)	0 (0%)	0 (0%)	0 (0%)
E. histolytica	0 (0%)	0 (0%)	0 (0%)	0 (0%)	0 (0%)	0 (0%)

Supplemental Table 3. Demographics and Health Characteristics by Diarrhea Type and PCR Status

**Patients Characteristic**	**All diarrhea patients (including without PCR and without data on blood in stool) (n = 1020)**	**Non-Bloody Diarrhea with PCR (n = 731)**	**Non-bloody diarrhea (including without PCR) (n= 860)**	**Bloody Diarrhea with PCR (n = 111)**	**Bloody Diarrhea (including without PCR) (n=126)**
Female gender	500 (49%)	342 (47%)	414 (48%)	59 (53%)	66 (52%)
**Race**					
White	532 (52%)	387 (53%)	446 (52%)	63 (57%)	66 (52%)
Black	240 (24%)	184 (25%)	214 (25%)	16 (14%)	19 (15%)
Other/Unknown	248 (24%)	160 (22%)	200 (23%)	32 (29%)	41 (32.5%)
**Ethnicity**					
Hispanic/Latino	293 (29%)	206 (28%)	249 (29%)	31 (28%)	39 (31%)
Non-Hispanic or Latino	694 (68%)	507 (69%)	586 (68%)	76 (68%)	81 (64%)
Not specified/refused	33 (3.2%)	18 (3%)	25 (2.9%)	4 (3.6%)	6 (4.8%)
**Age (Mean/Media)**	4.7/2.5	4.4/2.3	4.4/2.2	6.5/5.2	6.5/5
**Age Group**					
<6 months	113 (11%)	82 (11%)	97 (11%)	12 (11%)	12 (9.5%)
6-23 months	323 (32%)	255 (35%)	292 (34%)	23 (21%)	24 (19%)
2-4 years	239 (23%)	171 (23%)	204 (24%)	19 (17%)	25 (20%)
5-11 years	227 (22%)	152 (21%)	179 (21%)	34 (31%)	41 (33%)
12-17 years	118 (12%)	71 (10%)	88 (10%)	23 (21%)	24 (19%)

**Underlying Medical Condition**	273 (26%)	198 (27%)	233 (27.2%)	25 (23%)	29 (23%)
**Immunosuppressed**	9 (3%)	7 (3.5)	7 (3.0%)	1 (4%)	1 (3.4%)
**Insurance**					
Public	646 (63%)	469 (64%)	554 (64%)	62 (56%)	72 (57%)
Private	285 (28%)	192 (26%)	226 (26%)	43 (39%)	48 (38%)
None/Other	89 (9%)	70 (10%)	80 (9.3%)	6 (7%)	6 (4.7%)
**Attend pre-school or day care**	545 (53%)	393 (54%)	450 (52%)	70 (63%)	78 (61%)
**Pet or animal exposure**	581 (57%)	405 (55%)	482 (56%)	69 (62%)	79 (63%)
**International Travel (past month)**	22 (2%)	12 (2%)	14 (2%)	7 (6%)	8 (6%)
**Season**					
Summer (Jul-Sep)	301 (30%)	201 (27%)	255 (30%)	28 (25%)	36 (29%)
Fall (Oct-Dec)	271 (27%)	201 (27%)	229 (27%)	33 (30%)	34 (27%)
Winter (Jan-Mar)	244 (24%)	193 (26%)	209 (24%)	28 (25%)	31 (25%)
Spring (Apr-Jun)	204 (20%)	136 (19%)	167 (19%)	22 (20%)	25 (20%)

Supplement Table 4. Patients with bloody diarrhea and PCR who initiated antibiotics in or after the emergency department. “Pre-intervention” group refers to a period of the study where multiplex PCR results were not available to the clinician. “Intervention” group refers to a period of the study where multiplex PCR results were generally available in the electronic health record within approximately 1–4 hours and communicated to the clinician. Abbreviations: ED, emergency department, Abx, antibiotics

**Group**	**Age group**	**Pathogen detected**	**Abx start in ED**	**Abx start after ED**
Pre-Intervention*	5-12 years	None	None	Metronidazole
Intervention	2-4 years	*C. difficile*	Ceftriaxone	NA
Intervention*	6-23 months	STEC O157, astrovirus	None	Ciprofloxacin, Piperacillin-Tazobactam
Intervention	12-17 years	*C. difficile*	Metronidazole	Vancomycin, Fidaxomicin
Intervention*	5-12 years	None	None	Piperacillin-Tazobactam
Intervention	2-4 years	*Shigella/EIEC*	None	Azithromycin
Pre-Intervention	< 6 months	Rotavirus A, *Salmonella*	Ceftriaxone	None
Pre-Intervention*	5-12 years	STEC O157	None	Ciprofloxacin, Vancomycin, Metronidazole, Piperacillin-Tazobactam, Erythromycin
Pre-Intervention*	12-17 years	None	None	Metronidazole
Intervention*	5-12 years	*Salmonella*	Ceftriaxone	None
Intervention*	5-12 years	*C. difficile, Salmonella*	Ceftriaxone	None
Intervention	< 6 months	*Salmonella*	None	Ceftriaxone
Intervention*	6-23 months	None	None	Vancomycin, Metronidazole
Intervention	2-4 years	*Campylobacter*	Azithromycin	None
Pre-Intervention	2-4 years	*Campylobacter*	Cefdinir	None
Pre-Intervention*	12-17 years	None	Metronidazole	None
Pre-Intervention	5-12 years	*Shigella/EIEC*	Trimethoprim-Sulfamethoxazole	None
Pre-Intervention	5-12 years	*Shigella/EIEC*	Azithromycin	None
Pre-Intervention	5-12 years	*Shigella/EIEC*	Azithromycin	None
Pre-Intervention	5-12 years	*EAEC, Shigella/EIEC*	Trimethoprim-sulfamethoxazole	None
Intervention	12-17 years	*C. difficile,* norovirus	Metronidazole	None

Intervention*	< 6 months	sapovirus	Ceftriaxone, Vancomycin	None
Intervention	6-23 months	*Shigella/EIEC*	Azithromycin	None
Intervention	5-12 years	*Shigella/EIEC*	Azithromycin	None
Intervention	5-12 years	*Shigella/EIEC*	Azithromycin	None
Intervention	5-12 years	*EAEC, Shigella/EIEC*	Ciprofloxacin	None

Footnote: *Patients for which antibiotics are not indicated. 6 patients had no pathogen or only virus detected, 2 had *Salmonella* detected in non-infants and 2 had STEC.
